# Assistive diagnostic indicators for infections related to lumbar posterior interbody fusion internal fixation: platelet count and mean platelet volume

**DOI:** 10.1186/s13018-023-04358-4

**Published:** 2023-11-20

**Authors:** Yadong Zhang, Houkun Li, Wentao Wang, Lequn Shan, Dingjun Hao

**Affiliations:** 1 Department of Spine Surgery, Honghui Hospital, Xi’an Jiaotong University, Xi’an, 710054 Shaanxi China; 2 Graduate School, Xi’an Medical University, Xi’an, 710068 Shaanxi China; 3 Shaanxi Key Laboratory of Spine Bionic Treatment, Xi’an, 710054 Shaanxi China

**Keywords:** Deep surgical site infection, Diagnosis, Platelets, Interbody fusion internal fixation

## Abstract

**Background:**

The most severe complication after posterior single-segment lumbar interbody fusion and internal fixation (PIFIF) surgery for degenerative lumbar diseases is deep surgical site infection (DSSI). Preoperatively diagnosing such complications proves to be challenging. Platelets, as acute-phase reactants, undergo changes in response to infections and inflammation. This study aims to assess whether platelet indices can further aid in the diagnosis of DSSI.

**Methods:**

A single-center retrospective study was conducted from January 2016 to February 2021 at Xi'an Jiaotong University-Affiliated Honghui Hospital, involving 83 patients who underwent revision surgery after PIFIF due to lumbar degenerative diseases. Among them, 24 patients were diagnosed with DSSI based on combined bacterial culture and imaging data. Preoperative complete serological indicators including erythrocyte sedimentation rate (ESR), C-reactive protein (CRP), and platelet count and mean platelet volume ratio (P/M ratio) were analyzed using receiver operating characteristic (ROC) curve analysis to determine cutoff values, sensitivity, and specificity. This was done to further assess the ability of these serological indicators to identify the occurrence of DSSI after PIFIF.

**Results:**

There were no significant differences in baseline demographic characteristics between the two patient groups (*P* > 0.05). The P/M ratio was 13.54 ± 5.05 in the aseptic revision group, while it was 19.21 ± 6.30 in the DSSI revision patients, showing a significant difference (*P* < 0.001). ROC curve analysis revealed that the optimal cutoff value for the P/M ratio was 17.50, with a sensitivity of 58.3% and a specificity of 78.6%. The areas under the curve (AUC) for ESR, CRP, and P/M ratio were 0.797, 0.845, and 0.756, respectively. The negative predictive value (NPV) was 87.04%, 89.47%, and 82.45%, respectively; the positive predictive value (PPV) was 58.62%, 69.23%, and 53.84%, respectively, for ESR, CRP, and P/M ratio, respectively. When P/M ratio is used in combination with ESR and CRP, the AUC is 0.887, with a sensitivity of 95.4%, specificity of 67.8%, NPV of 97.56%, PPV of 54.76%. The diagnostic performance of the model for evaluating DSSI is significantly improved compared to using ESR and CRP alone (*P* < 0.05).

**Conclusion:**

Platelets and their related serum biomarkers are closely associated with DSSI. The P/M ratio can serve as a reliable test for screening DSSI and is worth considering for inclusion in the assessment of patients at risk of developing DSSI after potential PIFIF surgery.

## Introduction

The main surgical method for degenerative lumbar diseases is posterior single-segment lumbar interbody fusion internal fixation (PIFIF). One of the most serious postoperative complications is deep surgical site infection (DSSI). If not diagnosed or treated promptly, it can lead to catastrophic consequences such as systemic inflammatory response syndrome, sepsis, or even death, adding extra complexity to our patient assessment and treatment [1]. Despite various diagnostic measures available, including laboratory tests, imaging examinations, and pathogen cultures, preoperative serum biomarkers such as white blood cell count, erythrocyte sedimentation rate (ESR), and C-reactive protein (CRP) also play a crucial role in the diagnostic evaluation of these patients. However, some studies suggest that these markers lack diagnostic capability in reliably screening potential DSSI patients [2, 3]. Local puncture or intraoperative secretion bacterial culture is employed as a definitive diagnostic strategy, but finding rapid, cost-effective, and accurate diagnostic indicators remains challenging for many infection patients.

A considerable amount of prior research has elucidated the role of platelets in the inflammatory response of our bodies to bacterial invasion, demonstrating their ability to clear and gather microbial invaders in support of leukocyte function [4–8]. There are also a few studies that have explored the potential diagnostic role of platelets and related markers in limb fractures and joint surgical infections [9–12]. However, in the field of spine surgery, particularly regarding postoperative spinal fusion, there is scarce literature investigating the potential role of platelet indicators [13, 14]. Given the well-documented role of platelets in the systemic inflammatory response of our bodies, without imposing additional financial burden on patients, it becomes a worthwhile avenue to explore. Additionally, serum testing can be conducted in all patients, including those who are unable to undergo local puncture for bacterial culture. Therefore, the objective of this study is to determine whether two common laboratory indicators related to platelets, platelet count (PLT), and mean platelet volume (MPV), can serve as auxiliary tools in diagnosing suspected DSSI.

## Patients and methods

### Patients

A single-center retrospective study was conducted on 83 patients who underwent PIFIF surgery at the Spinal Hospital of Xi'an Jiaotong University-Affiliated Honghui Hospital from January 2016 to February 2021 due to degenerative diseases of the lumbar spine (including lumbar disc herniation (LDH), lumbar spinal stenosis (LSS), and degenerative lumbar spondylolisthesis (DLS)). Among them, 59 cases were aseptic revisions, and 24 cases experienced DSSI revisions. Inclusion criteria were as follows: (1) aseptic revision cases, including non-infectious factors such as loosening, fracture of internal fixation materials, metal toxicity, or unexplained pain; (2) DSSI revision cases, including clinical signs of surgical site infection, imaging evidence of infection, or positive bacterial culture of local secretions or wound exudates during the surgery. 3) DSSI patients had no systemic inflammatory response syndrome (SIRS) before the surgery, and SIRS was diagnosed by meeting two or more of the following clinical criteria: body temperature > 38℃ or < 36℃, peripheral blood leukocyte count > 12 × 10^9/L or < 4 × 10^9/L, immature white blood cells > 10%, heart rate > 90 beats per minute or systolic blood pressure < 90 mmHg, respiratory rate > 20 breaths per minute or hyperventilation (PaCO_2_ < 32 mmHg) [15, 16]. Exclusion criteria were as follows: (1) patients with a history of tumors, abnormal liver or kidney function, blood disorders, or immunodeficiency diseases; (2) prior use of antibiotics or other treatments; (3) acute or chronic infectious diseases and any history of blood transfusion within the past year for any reason; (4) use of anticoagulants or drugs-affecting platelets; (5) no other surgical treatment was performed during the initial operation and revision surgery.

### Laboratory assessment

All patients undergo peripheral venous blood collection before surgery, which is stored in tubes containing EDTA-K2 and sent to the laboratory for analysis using the Japanese Sysmex XN automated analyzer. C-reactive protein is measured using the latex-enhanced immunoturbidimetric method. All measurement results are reviewed by three expert biochemistry professors. If any of the values are abnormal, the corresponding measurement will be repeated. The reference values for blood tests in the laboratory of the Affiliated Honghui Hospital of Xi'an Jiaotong University are as follows: platelet count (125–350 × 10^9/L), mean platelet volume (9–13fL), erythrocyte sedimentation rate (0–15 mm/h), and C-reactive protein (0–5 mg/L).

### Statistical analysis

Statistical analysis was performed using SPSS version 26.0 (Chicago, USA). Continuous numerical variables were presented as Mean ± SD, and independent sample *t tests* were used for comparisons between the two groups. Categorical variables were represented by frequencies, and the comparisons between the two groups were made using the *Chi-square test* or *Fisher's* exact test. To clarify the predictive value of various hematological parameters for the diagnosis of DSSI, receiver operating characteristic (ROC) curve analysis was conducted using MedCalc version 20.0 (Ostend, Belgium) software. The area under the ROC curve (AUC), specificity, and sensitivity were calculated, and the optimal cutoff point with the highest Youden index was selected to maximize sensitivity and specificity. Additionally, the diagnostic performance of the combined ESR and CRP model was compared with the combined ESR, CRP, and P/M ratio model. Throughout the entire study, a significance level of 0.05 was used to assess statistical significance.

## Results

### General population information

In this study, a total of 83 patients who underwent PIFIF surgery for degenerative lumbar diseases were included for revision. Among the 59 cases in the aseptic revision group, the primary disease types were as follows: 25 cases of LDH, 21 cases of LSS, and 13 cases of DLS. There were 32 male patients and 27 female patients, with an average age of 56.86 ± 10.08 years and a body mass index (BMI) of 22.76 ± 2.01. The Charlson Comorbidity Index (CCI) [17] was 0.71 ± 0.45, with 32.20% of patients having a history of smoking and 12.25% having a history of alcohol consumption. In the DSSI revision group of 24 cases, the primary disease types were as follows: 8 cases of lumbar disc herniation, 12 cases of lumbar spinal stenosis, and 4 cases of degenerative lumbar spondylolisthesis. There were 10 male patients and 14 female patients, with an average age of 60.13 ± 10.23 years and a BMI of 23.55 ± 1.85. The CCI was 0.67 ± 0.48, with 25.00% of patients having a history of smoking and 8.33% having a history of alcohol consumption. The results of bacterial culture in 24 cases showed that the most common was Staphylococcus aureus in 10 cases, accounting for 41.67%, followed by Staphylococcus epidermidis in 7 cases, accounting for 29.17%, and Escherichia coli in 5 cases, accounting for 20.83%. There were 2 cases of Acinetobacter baumannii, accounting for 8.33%. There were no significant differences between the two groups in terms of gender, age, BMI, smoking history, alcohol consumption history, CCI, and disease types (*P* > 0.05). Refer to Table 1.

**Table 1:** The demographic information of all patients

	Aseptic (n = 59)	DSSI (n = 24)	*t*/*χ*^*2*^ values	P value
CCI	0.71 ± 0.45	0.67 ± 0.48	0.402	0.688
BMI(kg/m^2^)	22.76 ± 2.01	23.55 ± 1.85	− 1.656	0.102
Gender (male)	32(54.24%)	10(41.67%)	1.079	0.299
Age (years)	56.86 ± 10.08	60.13 ± 10.23	− 1.330	0.187
Smoking history (positive)	19(32.20%)	6(25.00%)	0.421	0.517
Alcohol history (positive)	9(15.25%)	2(8.33%)	0.236	0.627
Disease type			1.414	0.547
LDH	25	8		
LSS	21	12		
DLS	13	4		

*DSSI* deep surgical site infection, *CCI* Charlson Comorbidity Index, *BMI* body mass index, *LDH* lumbar disc herniation, *LSS* lumbar spinal stenosis, *DLS* degenerative lumbar spondylolisthesis

### Clinical outcomes

**Fig. 1:**
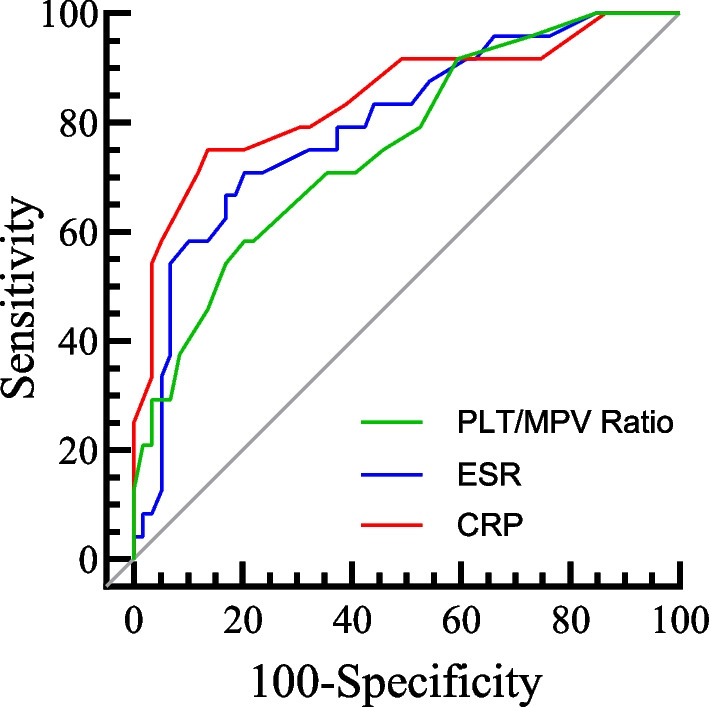
The ROC curves for ESR, CRP, and PLT/MPV ratio separately, with AUC of 0.797 for ESR, 0.845 for CRP, and 0.756 for PLT/MPV ratio. *ESR* erythrocyte sedimentation rate, *CRP* C-reactive protein, *AUC* areas under the ROC curves, *PLT* platelet count, *MPV* mean platelet volume; ROC, receiver operating characteristic

A study showed that the platelet count and platelet mean volume ratio (P/M ratio) of aseptic revision patients were 13.54 ± 5.05, while in DSSI revision patients, the P/M ratio was 19.21 ± 6.30, with a significant difference between the two (*P* < 0.001). ROC curve analysis for P/M ratio alone yielded AUC of 0.756. At the optimal ratio of 17.50, the sensitivity of this test would reach 58.3%, with a corresponding specificity of 78.6%. At this ratio, the negative predictive value (NPV) was 82.45%, and the positive predictive value (PPV) was 53.84%. Compared to the P/M ratio, the defined optimal values using ROC curve analysis for ESR and CRP showed higher specificity and sensitivity for both. The ROC curve analysis for ESR yielded an AUC of 0.797. At the optimal threshold of 37.00, ESR showed a sensitivity of 70.8%, specificity of 79.7%, NPV of 87.04%, and PPV of 58.62%. For CRP, the ROC curve analysis showed an AUC of 0.845, and at the optimal threshold of 15.50, CRP had a sensitivity of 75.0%, specificity of 86.4%, NPV of 89.47%, and PPV of 69.23%. Overall, there was no significant difference in the diagnostic specificity among the three individual markers, while in terms of sensitivity, the P/M ratio was slightly lower than ESR and CRP. However, all three markers exhibited high diagnostic performance, with AUC values greater than 0.7. Refer to Table 2 and Fig. 1 for details.

**Table 2:** Results of ROC analysis for serum biomarkers in the diagnosis of DSSI

	ESR(mm/h)	CRP(mg/L)	P/M ratio
Optimal threshold	37.00	15.50	17.50
Sensitivity	70.8%	75.0%	58.3%
Specificity	79.7%	86.4%	78.6%
AUC	0.797	0.845	0.756
Accuracy	77.11%	83.13%	73.49%
Positive likelihood ratio	3.487	5.515	2.724
Negative likelihood ratio	0.366	0.289	0.531
Positive predictive value	58.62%	69.23%	53.84%
Negative predictive value	87.04%	89.47%	82.45%
Standard error	0.0547	0.0525	0.0577
95%CI	0.689–0.904	0.742–0.948	0.642–0.869
Significance	< 0.001	< 0.001	< 0.001

*DSSI* deep surgical site infection, *ESR* erythrocyte sedimentation rate, *CRP* C-reactive protein, *AUC* areas under the ROC curves, *P*/*M* platelet count/mean platelet volume, *ROC* receiver operating characteristic

**Fig. 2:**
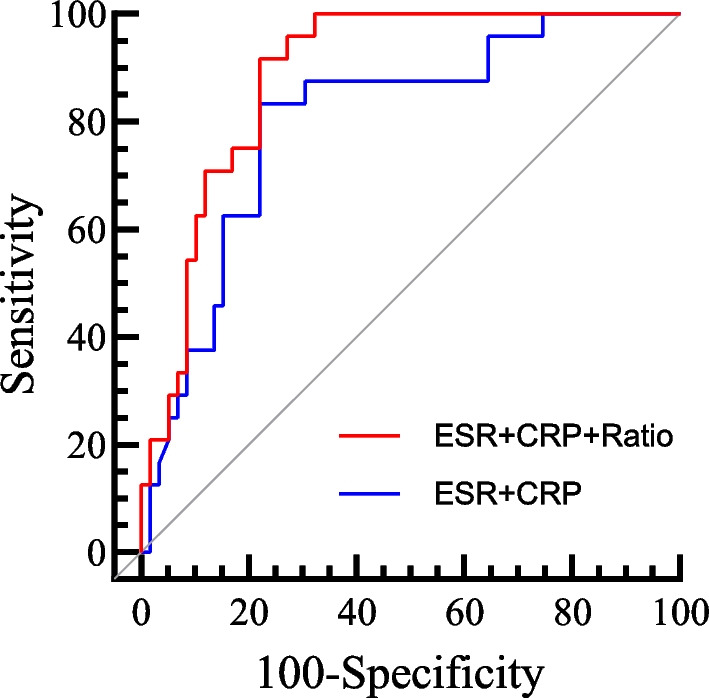
The AUC of the combined ROC curve for the two models of ESR and CRP was 0.803; the AUC of the combined ROC curve for the three models of ESR, CRP, and PLT/MPV ratio was 0.887. *ESR* erythrocyte sedimentation rate, *CRP* C-reactive protein, *AUC* areas under the ROC curves, *PLT* platelet count, *MPV* mean platelet volume, *ROC* receiver operating characteristic

We further conducted combined model ROC curve analysis to assess the diagnostic performance of ESR and CRP when combined with each other and with the P/M ratio. After merging the ESR and CRP models, the ROC curve analysis showed an AUC of 0.803, with corresponding sensitivity of 83.3%, specificity of 78.0%, negative predictive value of 92.00%, and positive predictive value of 60.61%. When analyzing the three models (ESR, CRP, and P/M ratio) together, the ROC curve analysis resulted in an AUC of 0.887, with corresponding sensitivity of 95.4%, specificity of 67.8%, NPV of 97.56%, and positive predictive value of 54.76%. The combined diagnostic performance of the three models showed a significant improvement in AUC compared to the analysis of the two models alone (*P* < 0.05). Refer to Table 3 and Fig. 2 for details.

**Table 3:** A comparison of the diagnostic performance of a combined model for diagnosing DSSI

	ESR + CRP	ESR + CRP + P/M ratio	P value
AUC	0.803	0.887	0.032
95%CI	0.700–0.905	0.818–0.956	
Sensitivity	83.3%	95.4%	
Specificity	78.0%	67.8%	
Accuracy	79.52%	75.90%	
Positive likelihood ratio	3.786	2.963	
Negative likelihood ratio	0.214	0.068	
Positive predictive value	60.61%	54.76%	
Negative predictive value	92.00%	97.56%	

*DSSI* deep surgical site infection, *ESR* erythrocyte sedimentation rate, *CRP* C-reactive protein, *AUC* areas under the ROC curves, *P*/*M*, platelet count/mean platelet volume

## Discussion

The PIFIF surgery is the primary surgical method for treating degenerative lumbar diseases. When postoperative infections are suspected, spine surgeons have been using ESR and CRP to preoperatively assess the likelihood of deep surgical site infection (DSSI) in patients. However, due to the lack of diagnostic reliability of these serological biomarkers, the current diagnostic gold standard still relies on bacterial cultures from locally secreted spinal fluid during surgery. This invasive and time-consuming detection method is expensive and is not widely used in most medical institutions. Therefore, in this retrospective study, we analyzed the practicality of two commonly available and easily obtainable laboratory indicators, PLT, and MPV, in diagnosing potential DSSI. The results suggest that when measured and analyzed using the P/M ratio, these indicators may serve as a more reliable screening test compared to traditional inflammatory serum markers.

Ideal serum biomarkers would accurately identify all patients with a certain disease and those without it, allowing for precise diagnosis without the need for further invasive tests. However, such a scenario is rarely encountered, and hence, the primary function of most serum biomarkers is screening, acting as a classification mechanism to identify individuals who require further diagnostic testing. To achieve this goal, serum biomarkers should correctly identify all individuals with a specific disease while allowing for a relatively low false-positive rate. A study has reported that patients with postoperative albumin below 30 g/L can increase the incidence of poor wound healing and thus the incidence of DSSI [18]. This may be due to the fact that albumin is widely involved in the body's immune process against external and self-pathogenic microorganisms, which indirectly improves one's own immunity. While analyzing the data of all the patients in this study, it was found that the preoperative and postoperative albumin of the patients was not less than 30 g/L, and it was then considered whether there was an indicator that could be used to assist in the diagnosis of DSSI. Therefore, in this retrospective study, we investigated the clinical utility of the P/M ratio as a potential diagnostic tool in the context of PIFIF after DSSI. We compared this ratio with more traditional inflammatory biomarkers like ESR and CRP. While platelets were traditionally considered merely incidental participants in the systemic inflammatory response to infection, mainly exerting their effects through the synthesis and secretion of inflammatory cytokines like interleukin-1 (IL-1), interleukin-6 (IL-6), and tumor necrosis factor (TNF), recent literature suggests their role is far more active than previously thought [19, 20]. Platelets are not only actively recruited to sites of inflammation and infection, but they can also directly or indirectly assist leukocytes in combating pathogens by employing microbial compounds [7, 21].

We chose platelets and related laboratory indicators for this study, first, because all patients need to undergo a complete blood cell count before surgery, which is easily accessible. Secondly, we know that platelets act as acute-phase reactants in the inflammatory immune response, playing an important role during systemic inflammation. In our study, we combined PLT and MPV measurements, mainly considering that during the period of systemic inflammatory response, as platelet reactions increase and megakaryocytes are influenced by high concentrations of platelet-derived cytokines, MPV tends to decrease [5]. This directional change in PLT and MPV leads to an increased ratio between the two, which may also explain the variability of platelet baselines in patients [22–24].

The research results show that the P/M ratio of patients in the DSSI group is significantly higher than that of the aseptic revision group, indicating a close correlation between the pathological status of DSSI and the P/M ratio. ROC curve analysis reveals that although the diagnostic performance AUC of the individual P/M ratio is slightly lower compared to ESR and CRP, it still exceeds 0.7, indicating a moderate diagnostic efficacy. Clinically, using a single laboratory index to assess DSSI is not reliable. Therefore, we combined the traditional inflammatory markers ESR, CRP, and P/M ratio as three biomarkers and found that the addition of the P/M ratio significantly increased the diagnostic performance AUC to 0.887, with a high sensitivity of 95.4%. The difference between the two methods also has statistical significance. Using a combination of multiple laboratory inflammatory parameters for assessing and identifying postoperative DSSI in lumbar vertebrae provides more confidence and higher accuracy. This finding is in line with our clinical practice.

Our research has some limitations. Firstly, it is a retrospective study, which means that our findings might be affected by selection bias. Although both groups in this study showed no differences in age, gender, BMI, CCI, smoking history, alcohol consumption, disease type, and baseline treatment, there are still other confounding factors that could influence our results. Secondly, the patient inclusion period for this study spanned 5 years, and the incidence rate of DSSI in our hospital is relatively low, leading to a small sample size. Therefore, future research will require large sample, multicenter, prospective studies to further validate our conclusions.

## Conclusions

The P/M ratio is closely related to the pathological changes of DSSI after PIFIF, making it a potentially reliable screening test for DSSI that is easily obtainable. Therefore, it is recommended that patients with lumbar degenerative diseases undergo both PIFIF and P/M ratio assessments to assist in diagnosing DSSI. Additionally, we can combine common laboratory indicators like ESR and CRP for a comprehensive evaluation of DSSI. Of course, clinical symptoms and imaging data should also be included in the assessment, allowing for a more confident diagnosis of DSSI without invasive examinations.

## Data Availability

The datasets used and/or analyzed during the current study are available from the corresponding author on reasonable request.

## Abbreviations

DSSI: Deep surgical site infection
PLT: Platelet count
MPV: Mean platelet volume
ESR: Erythrocyte sedimentation rate
CRP: C-reactive protein
AUC: Areas under the ROC curves
P/M: Platelet count/mean platelet volume
ROC: Receiver operating characteristic
PIFIF: Posterior single-segment lumbar interbody fusion and internal fixation
